# Synergism between the mTOR inhibitor rapamycin and FAK down-regulation in the treatment of acute lymphoblastic leukemia

**DOI:** 10.1186/s13045-016-0241-x

**Published:** 2016-02-18

**Authors:** Pei-Jie Shi, Lu-Hong Xu, Kang-Yu Lin, Wen-jun Weng, Jian-Pei Fang

**Affiliations:** Department of Pediatrics, Sun Yat-sen Memorial Hospital, Sun Yat-sen University, No. 107, West Yan Jiang Road, Guangzhou, Guangdong 510120 China; Department of Life Science, Sun Yat-sen University, No. 135, West Xin Gang Road, Guangzhou, Guangdong 510275 China

**Keywords:** Acute lymphoblastic leukemia, Focal adhesion kinase, Mammalian target of rapamycin inhibitor, Therapeutic efficacy, REH cells

## Abstract

**Background:**

Acute lymphoblastic leukemia (ALL) is an aggressive malignant disorder of lymphoid progenitor cells in both children and adults. Although improvements in contemporary therapy and development of new treatment strategies have led to dramatic increases in the cure rate in children with ALL, the relapse rate remains high and the prognosis of relapsed childhood ALL is poor. Molecularly targeted therapies have emerged as the leading treatments in cancer therapy. Multi-cytotoxic drug regimens have achieved success, yet many studies addressing targeted therapies have focused on only one single agent. In this study, we attempted to investigate whether the effect of the mammalian target of rapamycin (mTOR) inhibitor rapamycin is synergistic with the effect of focal adhesion kinase (FAK) down-regulation in the treatment of ALL.

**Methods:**

The effect of rapamycin combined with FAK down-regulation on cell proliferation, the cell cycle, and apoptosis was investigated in the human precursor B acute lymphoblastic leukemia cells REH and on survival time and leukemia progression in a non-obese diabetic/severe combined immunodeficiency (NOD/SCID) mouse model.

**Results:**

When combined with FAK down-regulation, rapamycin-induced suppression of cell proliferation, G_0_/G_1_ cell cycle arrest, and apoptosis were significantly enhanced. In addition, REH cell-injected NOD/SCID mice treated with rapamycin and a short-hairpin RNA (shRNA) to down-regulate FAK had significantly longer survival times and slower leukemia progression compared with mice injected with REH-empty vector cells and treated with rapamycin. Moreover, the B-cell CLL/lymphoma-2 (BCL-2) gene family was shown to be involved in the enhancement, by combined treatment, of REH cell apoptosis.

**Conclusions:**

FAK down-regulation enhanced the in vitro and in vivo inhibitory effects of rapamycin on REH cell growth, indicating that the simultaneous targeting of mTOR- and FAK-related pathways might offer a novel and powerful strategy for treating ALL.

## Background

Acute lymphoblastic leukemia (ALL) is an aggressive malignant disorder of lymphoid progenitor cells in both children and adults, and it is caused by genetic lesions in blood-progenitor cells [1, 2]. Although improvements in contemporary therapy and development of new treatment strategies [3–5] have led to dramatic increases in the cure rate in children, the relapse rate remains high and the prognosis of relapsed childhood ALL is poor [6]. Hematopoietic stem cell transplantation (HSCT) seems to be curative for patients with ALL relapse and high-risk acute lymphoblastic leukemia [7]. However, post-HSCT relapse is still an obstacle to therapeutic improvement [8, 9]. Use of an antineoplastic immunosuppressive agent after transplantation should facilitate treatment. However, although immunosuppressive therapy can significantly increase the survival time of transplant patients, it also promotes tumor growth [10].

Rapamycin, an inhibitor of mammalian target of rapamycin (mTOR), is a bacterial macrolide that was originally used as an antifungal agent [11, 12]. The findings that rapamycin targets mTOR and is also antiproliferative led to its use as an anticancer agent [10, 13]. Evidence indicates that the phosphatidylinositol 3-kinase (PI3K), Akt, mTOR signaling pathway (PI3K/Akt/mTOR) is dysregulated in hematologic malignancies and abnormally activated in childhood ALL. Most commonly, this abnormal activation is due to constitutive activation of Akt and provides a compelling rationale to target this pathway in ALL. Preclinical studies demonstrating significant activity against ALL has led to a number of clinical trials [14]. Combination therapeutic strategies of using rapamycin with focal adhesion kinase (FAK) down-regulation may address the problem of resistance to mTOR-targeted monotherapy and improve the treatment effect.

FAK is a 125-kDa non-receptor tyrosine kinase that plays an important role in cell survival, proliferation, apoptosis, migration, and invasion [15, 16]. FAK expression is higher in malignant cells than in the corresponding normal cells [17], and a high expression of FAK is associated with enhanced blast migration and poor prognosis in acute myeloid leukemia (AML) [18]. Furthermore, in our previous study, FAK down-regulation inhibited leukemogenesis in breakpoint cluster region/Abelson leukemia virus (BCR/ABL)-transformed ALL cells and increased apoptosis and drug efficacy in pro-B ALL cells. In vivo FAK down-regulation has also been shown to impair cell migration and inhibit leukemia progression [19]. Interestingly, FAK is an upstream kinase of Akt [20], indicating that FAK down-regulation might suppress rapamycin-induced Akt activation [21, 22]. This possibility provided us with a rationale for combining rapamycin with FAK down-regulation therapy to treat ALL in patients who received HSCT.

In the present study, we found that FAK was activated in tumor cells of ALL patients. Either FAK down-regulation or rapamycin caused growth inhibition of a pro-B ALL cell line, and growth was more profoundly inhibited by a combination of FAK down-regulation and rapamycin. The combination enhanced the treatment effect of rapamycin, prolonged median survival time, and slowed the progression of leukemia in non-obese diabetic/severe combined immunodeficiency (NOD/SCID) mice injected with REH (the human precursor B acute lymphoblastic leukemia cell line) cells. Collectively, our results suggest that the combination of rapamycin and FAK down-regulation may be a promising therapeutic strategy in ALL patients who received HSCT.

## Results

### FAK was highly expressed in leukemia cells of patients with ALL

**Fig. 1:**
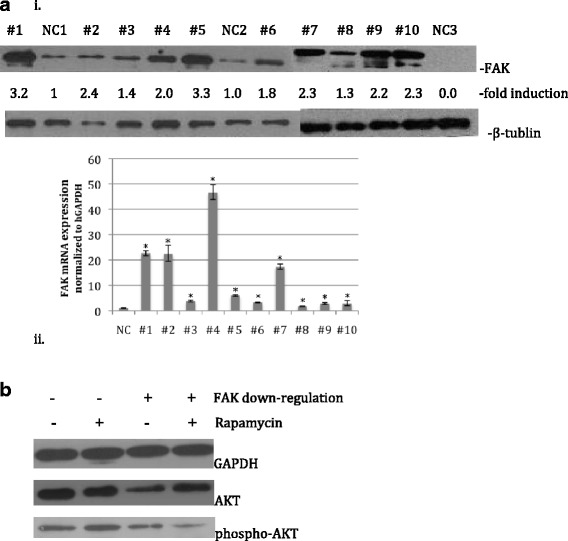
**a** FAK is activated in the cells of ALL patients. *i*: Western blot analysis. Cell lysates from freshly isolated ALL (*#1*–*#10*) and normal control volunteer (*NC1*–*NC3*) cells were prepared and subjected to Western blot analysis. The polyvinylidene fluoride membrane was sequentially probed with anti-FAK. The band intensity was measured via densitometry. *ii*: Quantitative real-time PCR analysis also showed that the mRNA expression of FAK was increased in ALL patients compared with normal control volunteers. The differences were statistically significant (*p* < 0.05). **b** Rapamycin activated AKT and FAK down-regulation inhibited AKT phosphorylation. After treatment with or without 100 nM rapamycin for 10 h, REH-empty vector cells or REH-FAK shRNA cells were subjected to Western blot analysis. AKT was activated by rapamycin, and phospho-AKT was inhibited by the combination treatment with rapamycin and FAK down-regulation

We examined whether FAK was activated in leukemia cells from patients and showed by Western blot analysis (Fig. 1a(i)) and confirmed by quantitative real-time polymerase chain reaction (PCR) analysis (Fig. 1a(ii)) that FAK protein expression is higher in leukemia cells from patients (*n* = 10) than in lymphocytes from normal volunteers (*n* = 3). The differences were statistically significant (*p* < 0.05).

### Rapamycin activated AKT and FAK down-regulation inhibited AKT phosphorylation

To examine whether FAK down-regulation was necessary for rapamycin-mediated effects, AKT and phospho-AKT levels were assayed to test our hypothesis. Western blot was used to analyze REH-empty vector cells or REH cells knocked down with FAK short-hairpin RNA (shRNA) and then treated with or without rapamycin 100 nM for 10 h. As shown in Fig. 1b, AKT was activated by rapamycin and phospho-AKT was inhibited by the combination of rapamycin and FAK down-regulation.

### FAK down-regulation enhanced the growth-inhibitory effects of rapamycin

**Fig. 2:**
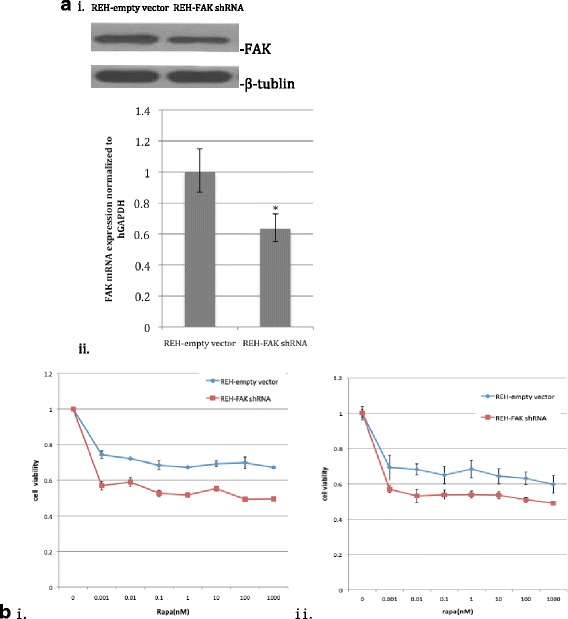
Targeting FAK via RNAi enhanced the growth-inhibitory effects of rapamycin. **a** FAK protein (*i*) and mRNA expression (*ii*) in REH-empty vector and REH-FAK shRNA cells. **b** The mTOR inhibitor rapamycin and FAK down-regulation inhibited the proliferation of REH cells. REH-empty vector or REH-FAK shRNA cells were plated in 96-well plates and cultured with rapamycin (0–1000 nM). After 2 days (*i*) or 3 days (*ii*), the cells were treated with CCK-8 (10 μl/well) for 2 h and the absorbance was measured. The results represent the mean ± S.D. of three experiments performed in triplicate

To study the effect of FAK down-regulation on rapamycin efficacy, FAK was down-regulated in REH cells by lentiviral-GFP-FAK-shRNA (demonstrated previously to block FAK expression specifically [23]). Western blot and quantitative real-time PCR analyses demonstrated that retrovirus-mediated RNAi successfully inhibited FAK protein expression and significantly inhibited mRNA expression (*p* < 0.05; Fig. 2a(ii)) by up to 50 % relative to the empty vector in REH cells.

Then, REH-empty vector and REH-FAK shRNA cells were plated in 96-well plates and cultured with rapamycin (0–1000 nM) for 48 or 72 h, respectively, after which cell numbers and viability were evaluated with the Cell Counting Kit-8 (CCK-8) assay. The combination of FAK down-regulation and rapamycin treatment induced enhanced growth inhibition of REH cells compared with rapamycin treatment alone. Rapamycin (100 nM, 48 h) alone inhibited the growth of REH cells by 30.19 ± 1.35 %, while the combination of FAK down-regulation and rapamycin inhibited growth by 50.68 ± 0.84 %. When these cells were exposed to rapamycin at the same concentration, the REH-FAK shRNA cells exhibited greater inhibition than the REH-empty vector cells. The results are shown in Fig. 2b.

### FAK down-regulation increased rapamycin-induced G_0_/G_1_ cell cycle arrest and cell apoptosis

**Fig. 3:**
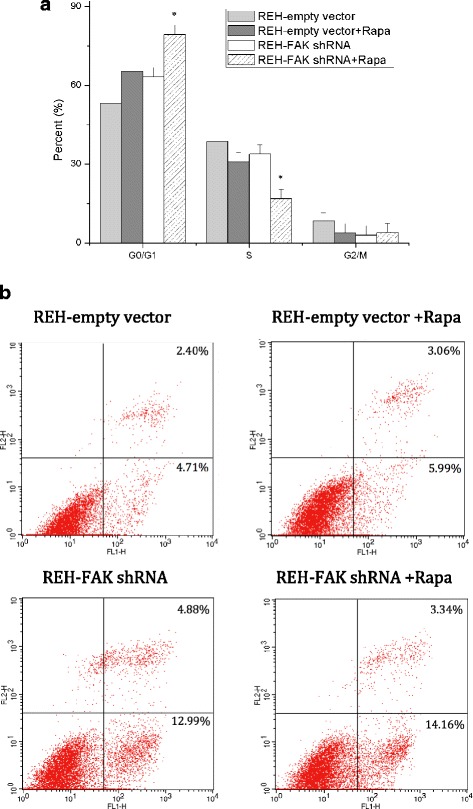
**a** The combination of rapamycin and FAK down-regulation induced enhanced G_0_/G_1_ cell cycle arrest in REH cells. REH-empty vector or REH-FAK shRNA cells were cultured with or without rapamycin (100 nM). After 48 h, the cell cycle was analyzed in these cells. The statistical significance of differences between populations in the G_0_/G_1_ and S phases of the cell cycle induced by either rapamycin or FAK down-regulation alone or by combined treatment with both was determined through one-way analysis of variance (ANOVA) followed by Bonferroni’s multiple comparison tests. The results represent the mean ± S.D. of three experiments performed in triplicate. *p < 0.05; Rapa, rapamycin. **b** The induction of apoptosis in REH cells by rapamycin and FAK down-regulation. REH-empty vector or REH-FAK shRNA cells were cultured with or without rapamycin (100 nM). After 30 h, cell apoptosis was analyzed using the annexin V/PI apoptosis detection kit. The combination of rapamycin and FAK down-regulation induced more cell apoptosis in REH cells than in rapamycin treatment alone

To reveal the mechanism underlying REH cell growth inhibition by the combined treatment, the cell cycle and cell apoptosis were examined. Marked accumulation of REH cells in G_0_/G_1_ phase occurred, with a concomitant decrease in the number of cells in the S phase after culture with rapamycin (100 nM, 48 h) or FAK down-regulation. Combined treatment further increased the percentage of cells in the G_0_/G_1_ phase and decreased that in the S phase (*p* < 0.05) (Fig. 3a). As shown in Fig. 3a, the percentages of cells in the G_0_/G_1_ cell cycle arrest in the rapamycin-only group and the combination group were 65.34 ± 3.62 % and 79.35 ± 3.28 %, respectively. The differences were statistically significant (*p* < 0.05). We next determined the effects of rapamycin and FAK down-regulation on cell apoptosis. REH-empty vector cells or REH-FAK shRNA cells were treated with or without 100 nM rapamycin for 30 h, stained with annexin V/propidium iodide (PI), and then analyzed by a flow cytometer. As shown in Fig. 3b, the percentage of annexin V^+^ cells was significantly higher after the combination treatment than rapamycin treatment only (17.50 ± 0.55 % vs 9.05 ± 0.41 %, respectively; *p* < 0.05), showing that the combined treatment was a more effective driver of cells into the G_0_/G_1_ cell cycle arrest and cell apoptosis.

### Effect of rapamycin, FAK down-regulation, and their combination on BCL-2 family mRNA expression in REH cells

**Fig. 4:**
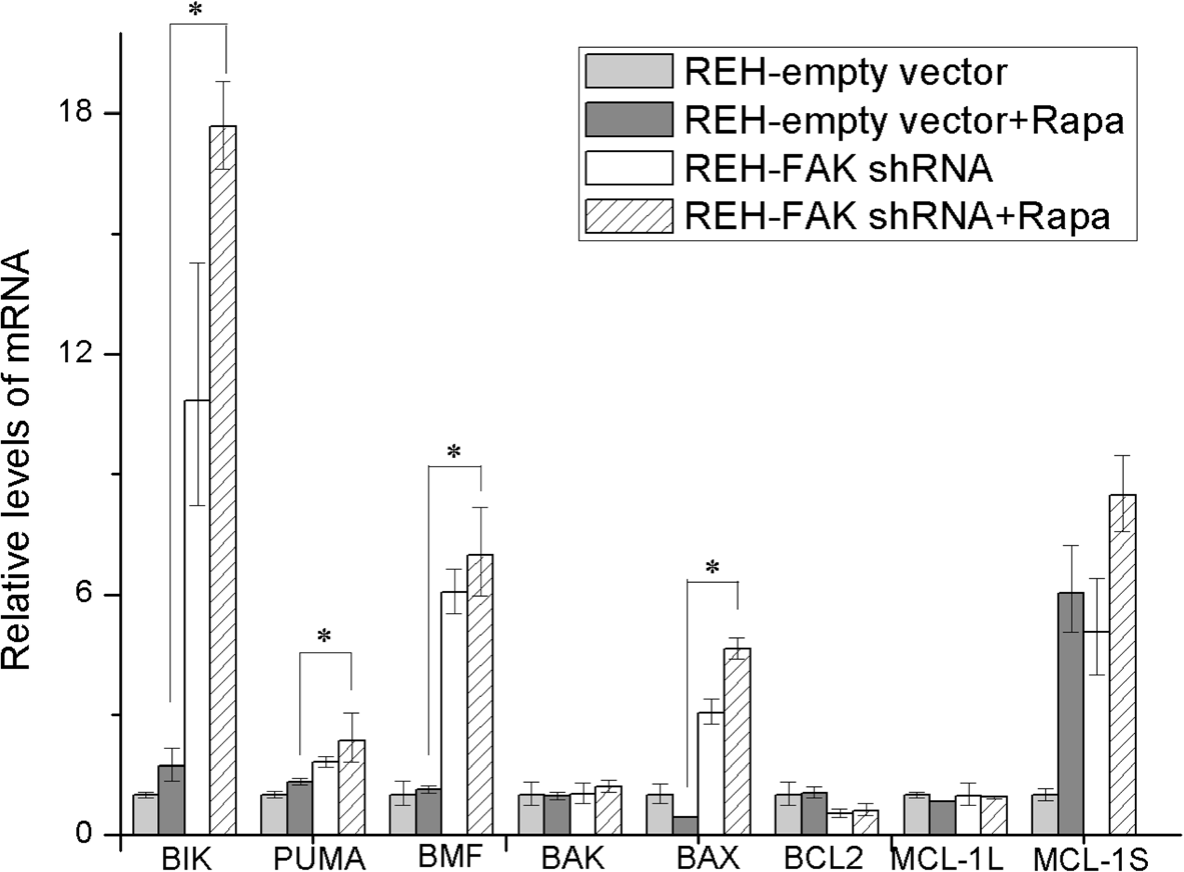
Changes in BCL-2 family expression caused by rapamycin treatment and FAK down-regulation. REH-empty vector or REH-FAK shRNA cells were cultured with or without rapamycin (100 nM). After 10 h, RNA was extracted from these cells and mRNA expression was detected using quantitative real-time PCR. The expression of pro-apoptosis genes, such as BIK, PUMA, BMF, BAX, and MCL-1S, was higher in the combination treatment group than in the rapamycin-only group. The expression of anti-apoptosis genes, such as BCL-2, was decreased in the combination treatment group compared with the rapamycin-only group. The results represent the mean ± S.D. of three experiments performed in triplicate. *p < 0.05; *Rapa* rapamycin

Subsequent experiments focused on the expression of apoptosis-related genes. B-cell CLL/lymphoma-2 (BCL-2) family mRNA expression was examined in REH-empty vector cells or REH-FAK shRNA cells treated with or without rapamycin 100 nM for 10 h using quantitative real-time PCR. As shown in Fig. 4, the expression of BCL-2 interacting killer (BIK) and other pro-apoptosis genes except BCL-2 antagonist killer (BAK) was significantly promoted by each treatment alone and significantly more by the combination therapy. Combined treatment resulted in a significantly higher mRNA expression of p53 up-regulated modulator of apoptosis (PUMA), BCL-2-modifying factor (BMF), and BCL-2-associated X protein (BAX) (*p* < 0.05), a lower mRNA expression of the anti-apoptosis gene BCL-2, and a 17.55-fold higher BCL-2/BAX ratio. However, expression of the pro-apoptosis fragment myeloid cell leukemia-1 (MCL-1)S was only slightly increased by the combination treatment relative to rapamycin alone.

### FAK down-regulation enhanced the in vivo efficacy of rapamycin

**Fig. 5:**
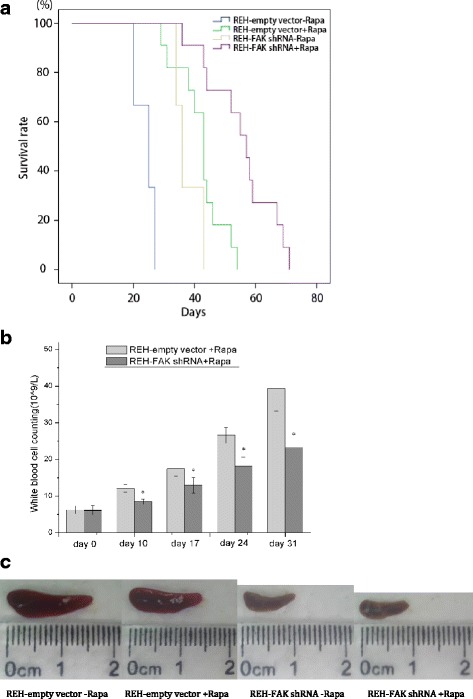
FAK down-regulation enhanced rapamycin efficacy in vivo. Five million REH-empty vector or REH-FAK shRNA cells were injected into NOD/SCID mice on day 0. The mice were treated daily with 1.5 mg/kg rapamycin or DMSO for 7 days beginning on day 10. The survivors were monitored daily, and leukemia progression was assayed on days 10, 17, 24, and 31. **a** Kaplan–Meier survival analysis showed that the combination of rapamycin and FAK down-regulation prolonged survival compared with rapamycin treatment alone. **b** The white blood cells in the peripheral blood were counted on days 0, 10, 17, 24, and 31. The results showed that the combination of rapamycin and FAK down-regulation reduced the leukemia burden compared with rapamycin treatment alone. *p < 0.05, the combination group vs. the rapamycin-only group. **c** Leukemic mice developed splenomegaly on day 25, but the combination of rapamycin and FAK down-regulation reduced the size of the spleens compared with rapamycin treatment alone

To further investigate the effects of FAK down-regulation on rapamycin efficacy in vivo, NOD/SCID mice were intravenously injected with REH cells (REH-empty vector cells or REH-FAK shRNA cells) and treated 10 days later with rapamycin 0.15 mg/kg for 7 days. All mice injected with REH cells died (Fig. 5a). With rapamycin treatment, death occurred between day 29 and day 52 with a median of 43 days (*n* = 11) in the REH-empty vector group and between day 43 and day 71 with a median of 57 days (*n* = 11) in the REH-FAK shRNA group. Without rapamycin treatment, the corresponding median survival times were 25 and 36 days, respectively (*n* = 3). Log-rank analysis showed a significant difference in median survival time between the treated empty vector and FAK shRNA groups (*p* < 0.05), indicating that FAK down-regulation prolonged the survival of rapamycin-treated NOD/SCID mice.

White blood cells (WBCs counted on day 0, day 10, day 17, day 24, and day 31 after REH cell injection) increased over time. Comparing the rapamycin-treated FAK shRNA group with the rapamycin-treated REH-empty vector group, the rise in WBC count was significantly slower (*p* < 0.01; Fig. 5b) and spleens removed from mice sacrificed on day 25 were much smaller in the rapamycin-treated FAK shRNA group (Fig. 5c), indicating that FAK down-regulation increases rapamycin-induced inhibition of leukemia progression in vivo.

It is intriguing that none of the 22 mice treated with rapamycin developed neurological disorders such as paralysis pressing one side of the head close to the floor or vertigo, indicating rapamycin treatment might prevent central nervous system leukemia. All the neurological disorders described above were present and severe in the three mice injected with the REH-empty vector without rapamycin treatment and were present and less severe in only one mouse injected with REH-FAK shRNA without rapamycin treatment.

## Discussion

In cancer therapy, combinatorial strategies are commonly used to treat malignancies that are resistant to standard treatment. Several combinations of rapamycin or rapalogs with other antitumor drugs have previously been investigated [24–26]. Recently, it was found that the cytotoxic drug carboplatin could enhance the effect of the mTOR inhibitor everolimus, and this combination is already undergoing phase I evaluation [27], which suggests that the clinical benefit from mTOR inhibitors could be maximized by combining them with other agents. In the present study, patients with ALL when compared to normal volunteers had varying degrees of FAK activation (Fig. 1a). In other studies, FAK was elevated in human AML, where increased FAK expression and activity were correlated with poor prognosis [18]. In our previous study, FAK down-regulation enhanced the drug efficacy of imatinib in ALL cells [19]. Herein, we assessed whether FAK could augment rapamycin efficacy in ALL cells, and the results were encouraging. In vitro, rapamycin combined with FAK down-regulation enhanced cell growth inhibition, G_0_/G_1_ cell cycle arrest, and cell apoptosis. In vivo, this combination slowed leukemia progression, indicating that FAK down-regulation can improve the efficacy of rapamycin in ALL treatment.

We supposed that rapamycin blocked the mTOR pathway, attenuating p70S6K activation and leading to Akt pathway activation. The antitumor activity of rapamycin is compromised by the hyperactivity of the feedback-loop-relevant PI3K/Akt signaling pathway [24]. Thus, when FAK is down-regulated, rapamycin-induced Akt phosphorylation is inhibited, which might explain how FAK down-regulation enhances the efficacy of rapamycin. The results in Fig. 1b strongly support this hypothesis.

The present finding that the blockade of mTOR by rapamycin and down-regulation of FAK inhibit proliferation of REH cells and increase G_0_/G_1_ cell cycle arrest (Figs. 2b and 3a) are consistent with the findings of other groups. Recher et al. showed that rapamycin could inhibit the proliferation of acute myeloid leukemia cells and sensitize these cells to growth inhibition mediated by cytotoxic agents such as cytarabine [28]. In another study, LY294002 was shown to enhance rapamycin-mediated inhibition of T-cell proliferation [29]. On the other hand, Pan et al. found that Caco-2 cell proliferation was significantly decreased by inhibition of FAK gene expression [30]. These results support our observations.

In our analyses, the induction of ALL cell apoptosis by rapamycin was mild. However, when rapamycin was combined with FAK down-regulation, the cell apoptosis effect was dramatically enhanced (Fig. 3b), indicating that the enhancement by FAK down-regulation of rapamycin’s antitumor efficacy is mainly due to apoptosis induction. Rapamycin treatment and/or FAK down-regulation can stimulate the expression of the pro-apoptosis genes, such as BIK, PUMA, BMF, BAX, and MCL-1S, and inhibit the expression of the anti-apoptosis genes such as BCL-2 and BCL-XL (Fig. 4). We suppose that using the new BCL-2 inhibitors in combination therapy will boost treatment efficacy [31].

Using a murine model of leukemia induced by REH cells, we further investigated the effects of FAK down-regulation on rapamycin efficacy in vivo. All the mice died after injection of leukemic cells despite treatment with rapamycin for 7 days. But compared with rapamycin treatment alone, rapamycin combined with FAK down-regulation prolonged median survival by 14 days, reduced spleen size, and diminished peripheral leukocyte count in the model mice. These findings suggest that FAK down-regulation potentiates rapamycin-induced inhibition of leukemia progression in vivo.

## Conclusions

The present study reported a new ALL therapy involving FAK down-regulation combined with the mTOR inhibitor rapamycin. FAK down-regulation was found to potentiate rapamycin-induced suppression of ALL cell growth both in vitro and in vivo, suggesting a new concept of ALL treatment (i.e., the targeting of both mTOR- and FAK-related pathways) to achieve more powerful therapeutic effect. Since the lentivirus-vector delivery system is not practical for clinical use, we suggest the testing of FAK inhibitors in future experiments [32, 33]. Because the precise mechanism is still unclear, the interaction between mTOR and FAK pathways should be explored in the future.

## Methods

### Cell culture and clinical samples

The REH human acute lymphoblastic pro-B cell leukemia cell line (CRL8286, ATCC, USA) was cultured in RPMI1640 medium (Gibco, USA) supplemented with 10 % fetal bovine serum (Gibco, USA) in a CO_2_ incubator. A total of 13 clinical samples, including samples from 8 patients with a primary diagnosis of acute lymphoblastic leukemia (ALL), 2 patients with relapsed ALL, and 3 normal samples from Sun Yat-sen Memorial Hospital, were included in this study. All cells were freshly isolated from the bone marrow of each individual. The clinical characteristics of these individuals are presented in Table 1. All patients provided informed consent, and the study was approved by the ethics committees of Sun Yat-sen Memorial Hospital.

**Table 1:** Patient clinical characteristics

Patient	Age (years)	Sex	Disease status
#1	12	Male	ALL newly diagnosed
#2	3	Female	ALL newly diagnosed
#3	2	Male	ALL newly diagnosed
#4	7	Female	ALL newly diagnosed
#5	9	Female	ALL newly diagnosed
#6	7	Female	ALL newly diagnosed
#7	7	Female	ALL newly diagnosed
#8	8	Male	ALL newly diagnosed
#9	15	Male	ALL relapse
#10	13	Female	ALL relapse
NC1	14	Female	Normal control
NC2	15	Male	Normal control
NC3	7	Male	Normal control

*ALL* acute lymphoblastic leukemia, *NC* normal control

Down-regulation of FAK with shRNA and establishment of stable transfected clones.

A short-hairpin RNA (shRNA)-expressing lentivirus-vector delivery system was applied as previously described [34, 35]. The obtained lentiviruses, containing the GFP-FAK shRNA vector or a GFP-empty vector construct, were used for the transfection of REH cells. To establish stable transfected clones, the REH cells were sorted repeatedly based on a green fluorescent protein (GFP) expression using a flow cytometer (FACSAria, Becton Dickinson, CA) at 72 h after transfection, until the percentage of GFP-positive clones was greater than 99 %. The stably transfected clones were used for further experiments. Quantitative real-time PCR analysis revealed that the best silencing efficiency was achieved with the shRNA designated FAK X40-2 shRNA, and the FAK target sequence was 5′-GGAATGCTTCAAGTGTGCTT-3′.

### Reagents

Rapamycin, a mammalian target of rapamycin (mTOR) inhibitor, was purchased from Sigma (USA). Rapamycin was dissolved in 100 % dimethyl sulfoxide (DMSO) (Sigma, USA) to a stock concentration of 25 mg/ml and stored at −20 °C.

### Western blotting and quantitative real-time PCR

The cells were lysed in radio immuno-precipitation assay (RIPA) buffer (Pierce, Rockford, IL, USA) with protease and phosphatase inhibitors (Roche, Beijing, China), and the supernatant was collected after centrifugation. Denatured proteins were fractionated via electrophoresis on a 10–12 % sodium dodecyl sulfate (SDS) polyacrylamide gel and transferred to a methanol-activated polyvinylidene fluoride (PVDF) membrane (Millipore). The membrane was blocked for 2 h in Tris-buffered saline Tween-20 (TBST) containing 5 % bovine serum albumin and then incubated with a polyclonal mouse anti-FAK (Millipore, USA), rabbit anti-AKT (Cell Signaling Technology, Boston, MA, USA), rabbit anti-phospho-AKT (Ser473, Cell Signaling Technology, Boston, MA, USA), rabbit anti-GAPDH (Cell Signaling Technology, Boston, MA, USA), or rabbit anti-β-tubulin (Cell Signaling Technology, Boston, MA, USA) antibody overnight at 4 °C. One hour after incubation with the corresponding goat anti-mouse (Thermo) or goat anti-rabbit (Sigma) horseradish peroxidase-conjugated secondary antibody, the level of protein expression was detected using the enhanced chemiluminescence (ECL) method (Millipore, USA) according to the manufacturer’s instructions.

Total RNA was extracted using the TRIzol reagent (Invitrogen, USA) according to the manufacturer’s protocols. cDNA was prepared from 1 μg of total RNA using a reverse transcription-polymerase chain reaction (RT-PCR) kit (Takara, Japan) with oligodT according to the manufacturer’s instructions. cDNA samples were then analyzed via quantitative real-time PCR using SYBR Green (Takara, Japan) in an ABI Step One Real-Time PCR machine (Applied Biosystems, Foster City, CA), with 40 cycles of 95 °C for 15 s and 60 °C for 30 s. The efficiency of cDNA synthesis was estimated using hGAPDH as a house-keeping gene. All data were analyzed via the comparative C_T_ method [36], and all of the reactions were performed in triplicate.

### Cell proliferation assays

REH-empty vector or REH-FAK shRNA cells (4 × 10^5^/ml) were incubated with various concentrations of rapamycin for 48 or 72 h, respectively, in 96-well plates (Costor, USA). After culture, Cell Counting Kit-8 (CCK-8, Dojindo Molecular Technologies, Shanghai, China) solution (10 μl) was added to each well, followed by incubation at 37 °C for an additional 2 h. The absorbance was measured at 450 and 630 nm using an absorbance reader. All experiments were performed in triplicate and were repeated at least three times.

### Cell apoptosis and cell cycle analysis

Apoptosis was assessed through annexin V/propidium iodide (PI) staining. After transduced REH cells were treated with or without rapamycin (100 nM) for 30 h, they were stained with annexin V/PI (BD Biosciences, CA, USA) following the manufacturer’s instructions and then analyzed via flow cytometry. Cell cycle analysis was performed on transduced REH cells incubated with or without rapamycin (100 nM) for 48 h. The cells were subsequently collected and incubated with 0.5 ml of NP40/PI buffer and RNase (25 μg/ml) for 30 min at 37 °C. Analysis by flow cytometry was performed immediately thereafter.

### In vivo experiments

Male NOD/SCID mice were purchased from the Huafukang Company (Beijing, China) and were maintained in the animal center of Sun Yat-sen University under specific pathogen-free conditions. The murine model of leukemia was established as previously described [19]. Briefly, five million REH-FAK shRNA or REH-empty vector cells were injected into 6- to 8-week-old NOD/SCID mice via the tail vein. For the in vivo assessment of drug effects, 10 days after transplantation of the REH cells, the mice were treated daily with 1.5 mg/kg rapamycin (or DMSO for the control group) via intraperitoneal injection for 7 days. Survival was monitored daily. Full blood counts were performed once a week. Several mice were sacrificed 25 days after transplantation, and their spleens were removed. The animal study was approved by the ethics committees of the animal center of Sun Yat-sen University.

### Statistical analysis

The results are expressed as the mean ± standard deviation (S.D.). The differences between groups were analyzed using Student’s *t* test when only two groups were compared or one-way analysis of variance (ANOVA) when more than two groups were compared. Log-rank *p* values were determined using the Kaplan–Meier method comparing survival curves. Values of *p* ≤ 0.05 were considered statistically significant.

## Abbreviations

ALL: acute lymphoblastic leukemia
AML: acute myeloid leukemia
BAK: BCL-2 antagonist killer
BAX: BCL-2-associated X protein
BCL-2: B-cell CLL/lymphoma-2
BCR/ABL: breakpoint cluster region/Abelson leukemia virus
BIK: BCL-2 interacting killer
BMF: BCL-2-modifying factor
CCK-8: Cell Counting Kit-8
DMSO: dimethyl sulfoxide
ECL: enhanced chemiluminescence
FAK: focal adhesion kinase
GFP: green fluorescent protein
HSCT: hematopoietic stem cell transplantation
MCL-1: myeloid cell leukemia-1
mTOR: mammalian target of rapamycin
NOD/SCID: non-obese diabetic/severe combined immunodeficiency
PCR: polymerase chain reaction
PI: propidium iodide
PI3K: phosphatidylinositol 3-kinase
PUMA: p53 up-regulated modulator of apoptosis
PVDF: polyvinylidene fluoride
RIPA: radio immuno-precipitation assay
SDS: sodium dodecyl sulfate
shRNA: short-hairpin RNA
TBST: Tris-buffered saline Tween-20
WBC: white blood cell
